# Local hyperthermia for esophageal cancer in a rabbit tumor model: Magnetic stent hyperthermia versus magnetic fluid hyperthermia

**DOI:** 10.3892/ol.2013.1618

**Published:** 2013-10-11

**Authors:** JIAYI LIU, NING LI, LI LI, DANYE LI, KAI LIU, LINGYUN ZHAO, JINTIAN TANG, LIYA LI

**Affiliations:** 1 Department of Oncology, Xiangya Hospital, Central South University, Changsha, Hunan 410008, P.R. China; 2 Department of Engineering Physics, Institute of Medical Physics and Engineering, Key Laboratory of Particle and Radiation Imaging, Ministry of Education, Tsinghua University, Beijing 100084, P.R. China; 3 Department of Pathology, The People’s Hospital of Zhangqiu, Zhangqiu, Shandong 250200, P.R. China; 4 Department of Medical Oncology, China-Japan Friendship Hospital, Beijing 100029, P.R. China; 5 Department of Stomatology, Second Hospital Affiliated to Jiamusi University, Jiamusi, Heilongjiang 154002, P.R. China

**Keywords:** magnetic mediated hyperthermia, esophageal cancer, magnetic nanoparticles, esophageal stent, alternative magnetic field

## Abstract

Magnetic-mediated hyperthermia (MMH) is a promising local thermotherapy approach for cancer treatment. The present study investigated the feasibility and effectiveness of MMH in esophageal cancer using a rabbit tumor model. The therapeutic effect of two hyperthermia approaches, magnetic stent hyperthermia (MSH), in which heat is induced by the clinical stent that is placed inside the esophagus, and magnetic fluid hyperthermia (MFH), where magnetic nanoparticles are applied as the agent, was systematically evaluated. A rabbit esophageal tumor model was established by injecting VX2 carcinoma cells into the esophageal submucosa. The esophageal stent was deployed perorally into the tumor segment of the esophagus. For the MFH, magnetic nanoparticles (MNPs) were administered to the rabbits by intratumoral injection. The rabbits were exposed under a benchtop applicator using an alternative magnetic field (AMF) with 300 kHz frequency for the hyperthermia treatment. The results demonstrated that esophageal stents and MNPs had ideal inductive heating properties upon exposure under an AMF of 300 kHz. MSH, using a thermal dose of 46°C with a 10-min treatment time, demonstrated antitumor effects on the rabbit esophageal cancer. However, the rabbit esophageal wall is not heat-resistant. Therefore, a higher temperature or longer treatment time may lead to necrosis of the rabbit esophagus. MFH has a significant antitumor effect by confining the heat within the tumor site without damaging the adjacent normal tissues. The present study indicates that the two hyperthermia procedures have therapeutic effects on esophageal cancer, and that MFH may be more specific than MSH in terms of temperature control during the treatment.

## Introduction

Esophageal cancer is a major health burden that constitutes one of the major causes of cancer-related mortality worldwide (1). The incidence of esophageal cancer has increased considerably during the past decades. A recent report from the GLOBOCAN project indicated that ~481,000 new cases and ~406,000 mortalities occurred globally during 2008, while China was estimated to have 258,000 new cases and ~210,000 mortalities, accounting for 53.6% of the new cases and 51.7% of the mortalities worldwide, respectively (2). Currently, the gold standard approach to treating early stage esophageal cancer has been to perform an esophagectomy, either alone or in combination with chemotherapy and/or radiotherapy (3). Although the survival of patients has been shown to improve, this approach has been reported to have significant risks. With the exception of the risks associated with esophagectomy in early-stage esophageal cancer treatment, >65% of all esophageal cancers are incurable at diagnosis (4). These two factors may account for the poor five-year survival rate of esophageal cancer, which has been shown to be <20%, remaining one of the lowest for all cancers (5). Therefore, a safer and more effective medical treatment strategy for esophageal cancer is required.

For decades, hyperthermia has been recognized as an effective approach for various cancers, and has been shown to play a significant role in multimodal concepts for cancer treatment (6). Hyperthermia has unique advantages, and the biological effectiveness of heat in treating cancer has been fully recognized for decades. However, in clinical oncology, hyperthermia is currently regarded as the fourth line of therapy and is mainly applied as an adjunct, ranked below surgery, chemotherapy and radiotherapy (7). The technical challenges that are associated with the currently available hyperthermia modalities mainly include the difficulty of uniform heating within the tumor region until the required temperature is reached, without damaging the adjacent normal tissues (8). The recent breakthrough in magnetic-mediated hyperthermia (MMH) may bring new alternatives for cancer locoregional hyperthermia by confining the heat to within the tumor site (9).

Based on the mechanism of heating ferromagnetic agents under an alternative magnetic field (AMF), marked progress has been made in MMH research and in clinical oncology. For the treatment of esophageal cancer, previous studies have shown that magnetic stent hyperthermia (MSH) may be a safe and effective strategy that is able to combine MMH with stent placement for patients with inoperable esophageal cancer (10–14). The medical nickel-titanium (Ni-Ti) stent has been demonstrated to possess excellent inductive heating characteristics under an AMF (11–14). *In vitro* investigations on human esophageal squamous carcinoma ECA-109 cells demonstrated that MSH has an inhibitory effect on cell viability and that such an effect is dependent on the thermal dose (13).

With the explosive growth of nanotechnology, magnetic nanothermotherapy, also termed magnetic fluid hyperthermia (MFH) or magnetic nanoparticles (MNPs) hyperthermia (MNH), has been making rapid progress (15). The research output of MFH has been successfully applied in clinical oncology (16). In 2010, MFH received European regulatory approval as a primary treatment for brain cancer (17). Although MFH has been widely studied in the treatment of diseases, including prostate cancer (18), glioblastoma multiforme (19) and pancreatic (20), breast (21), liver (22) and lung (23) cancer, no insight has been reached on the therapeutic effect in esophageal cancer. In the present study, MNPs were synthesized using the chemical co-precipitation procedure and then the particles were modified by 3-aminopropyltriethoxysilane (APTES), a type of aminosilane. The antitumor effect of the fabricated MNPs as mediators for MFH was evaluated on a rabbit tumor model. Furthermore, the safety and advantages of MSH and MFH on rabbit esophageal cancer treatment were compared. The results may provide useful information for further elucidating the therapeutic effect of magnetic hyperthermia in esophageal cancer treatment. Deductions from the experimental observations may have clinical significance for the future application of magnetic hyperthermia to treat esophageal cancer.

## Materials and methods

### MNPs, esophageal stenting, application of AMF and temperature measurements

**Figure 1:**
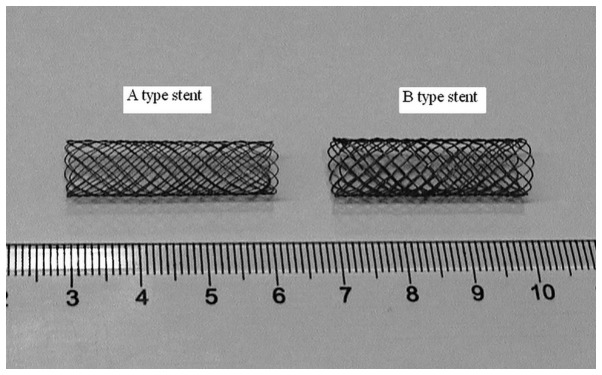
The two types of esophageal stents that were used in the present study.

**Figure 2:**
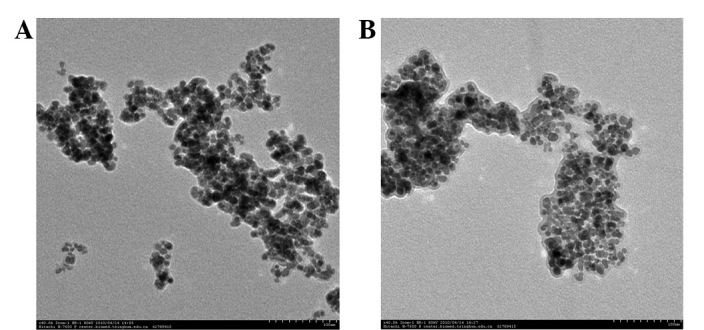
Transmission electron microscopy images of MNPs. (A) Uncoated MNPs. (B) APTES-coated MNPs. MNP, magnetic nanoparticles; APTES, 3-aminopropyltriethoxysilane.

APTES-coated MNPs, with diameters of 8–10 nm, were prepared using the co-precipitation of ferrous salts (FeCl_2_•4H_2_O and FeCl_3_•6H_2_O) by the addition of excess ammonium hydroxide as described previously (24). The morphology of the MNPs was observed using transmission electron microscopy (H-800; Brookhaven Instruments Corp., Holtsville, NY, USA) and the images are shown in Fig. 1. The superelastic nitinol stents (Grinkin Advanced Materials Co., Ltd., Beijing, China), which are made of Ni-Ti alloy, were of the same composition as the clinical esophageal stent. The stents were specially designed for the rabbit esophagus, with a length of 30 mm and a macroscopic diameter of 8 mm. Two types of stents, which were the same in diameter but with a difference in weight, were employed in the present study. The lighter stent (type A) was ~0.15 g, while the heavier stent (type B) was 0.29 g (Fig. 2).

**Figure 3:**
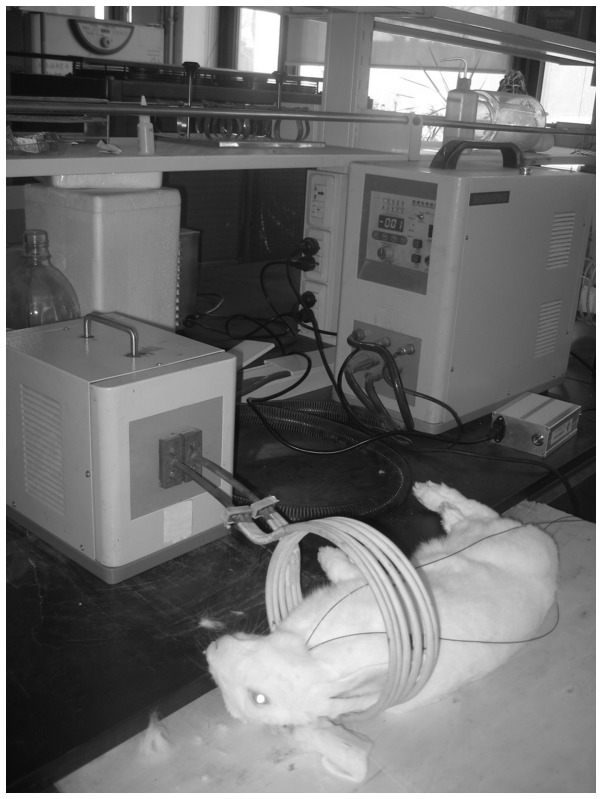
Experimental apparatus of the magnetic hyperthermia system (MHS).

The portable inductive heating device with 300 kHz and an adjustable field intensity was provided by Shuangping Instrument Technology, Co., Ltd. (Shenzhen, China). The field generator consisted of an alternating current generator feeding the coil inductor. The diameter of the coil was large enough to place the rabbit inside (Fig. 3).

A thermal-couple temperature probe (model IT-18 Copper-Constantan; Physitemp, Clifton, NJ, USA) was used for the temperature measurements. The probe fibers were connected to a four-channel millivoltmeter (model XSOL-4; Beijing Kunlun Tianchen Instrument Technology, Co., Ltd., Beijing, China) and the data were collected every 6 sec using a PC with home-written software. Prior to each experiment, calibration of the thermocouple was performed at 0 and 100°C.

### Inductive heating properties of esophageal stent and MNPs under AMF

For heating the stent, the thermocouple probe was fixed at the surface of the stent by inserting it to the mesh of the device. Following this, the thermocouple loaded stent was wrapped carefully by thermal insulation materials, including asbestos fibers, and placed into a water jacket incubator. The incubator was designed for temperature maintenance. The double-layer jacket was connected with a water bath so that the temperature inside the jacket was adjustable and could be maintained at ~37°C. The jacket was made of glass so the device itself did not induce heat when exposed under the AMF.

To investigate the inductive heating properties of the MNPs, a series of MNP suspensions of various particle concentrations were prepared by dispersing the MNPs using PBS. For taking the measurements, 2 ml of each suspension was carefully pipetted into an eppendorf tube. Prior to placing the tube inside the water jacket incubator, the thermocouple probe was placed inside the tube with the tip immersed under the suspension interface.

### Animal care and establishment of rabbit esophageal cancer

The maintenance and care of all the experimental animals that were used in this study was performed according to guidelines of the Institutional Animal Care and Use Committee of Tsinghua University (Beijing, China). Japanese white rabbits, each weighing ~2 kg, were provided by the Beijing Center for Disease Control and Prevention (Beijing, China). The rabbits were maintained in a specific pathogen-free animal house under a 12-h light and 12-h dark cycle and were fed a standard laboratory diet and tap water *ad libitum*.

The frozen VX2 tumor cells were thawed in a 37°C water bath and washed twice with PBS followed by centrifugation at 560 × g for 5 min. The viable cells were counted by trypan blue dye and re-suspended at 1×10^6^ cells/ml in PBS. A 1-ml viable cell suspension was injected into the thigh subcutis of a host rabbit to obtain the tumor node. The node was extracted when the diameter reached 3–4 cm. Furthermore, the grossly necrotic tissues in the tumor center and the surrounding myofascial tissue were discarded. The viable VX2 tissue was finely minced into fragments and ground in Dulbecco’s modified Eagle’s medium (DMEM). The cellular suspension was filtered through a sterile 74-μm mesh filter. The filtered cell suspension was re-suspended with DMEM at 1×10^8^ cells/ml.

The rabbits were anesthetized with i.v. 2% sodium pentobarbital into an ear vein. Under aseptic conditions, the rabbits were placed in a supine position and the esophagus region was shaved and prepared with iodophors and 75% alcohol. A 4–5-cm midline vertical incision was made along the trachea to expose the esophagus. A 0.1-ml VX2 tumor cell suspension, as mentioned previously, which contained 1×10^7^ viable cells was injected from the esophageal tunica adventitia into the submucosa of the cervical esophagus. The tumor was measured using a digital caliper. The tumor volumes (V) were determined by applying the following formula: V = a + b^2^, where a and b represented the maximal and minimal tumor diameters, respectively.

### Safety evaluation of MSH on the rabbit esophagus

Three levels of temperature (43, 46 and 50°C) were adopted for the safety evaluation of MSH on the rabbit esophagus. For each temperature, the rabbits underwent a 10- or 30-min MSH treatment. Therefore, six groups of rabbits were involved in this analysis (n=10). Following each treatment, the rabbits were routinely maintained for one week and then sacrificed for the histological analysis.

### Experimental groups for MSH and MFH

For the MSH treatment, the tumor-bearing rabbits were divided into two groups, the control (rabbits with stent implantation only) and MSH (n=6) groups. Three experimental groups, including the control (untreated group), MF (rabbits with esophageal tumor site infused with MNPs only) and MFH groups were involved in the MFH treatment.

### Stent deployment and MNP injection

The stents were deployed into the cervical esophagus using a stent delivery device. In order to monitor the temperature around the rabbit esophagus, the thermocouple probe was inserted into the stent mesh. As the stent has a good shape memory, it is easily deformed and inserted into the delivery device. Under X-ray, the stent loaded delivery device was inserted perorally and positioned precisely into the rabbit esophagus. The stent was then released and expanded to its original shape to impose a suitable force on the internal wall of the esophagus. A detailed illustration on the stent delivery device may be referred to in our previous publication (12).

Following the confirmation for the successful establishment of the rabbit tumor model by administration of a barium meal, the rabbits underwent an open surgical exposure of the esophagus. The colloidal liquid of aminosilane-coated MNPs, which dispersed with PBS, was administered to the tumor tissue by direct injection. In order to achieve a homogenous distribution of the MNPs within the tumor tissue, the intratumoral injection was performed from four directions, all of which were through the pinhole with a 27G syringe.

### Histological examination

Following the sacrifice of the rabbits, the esophagus, tumor and other tissues or organs were fixed in a 10% formalin solution. The tissue samples were paraffin-embedded, cut into 4-μm sections and further subjected to HE staining.

### Statistical analysis

A one-way analysis of variance was used for the statistical analysis. P<0.05 was considered to indicate a statistically significant difference.

## Results

### Inductive heating properties of esophageal stent and MNPs under AMF

**Figure 4:**
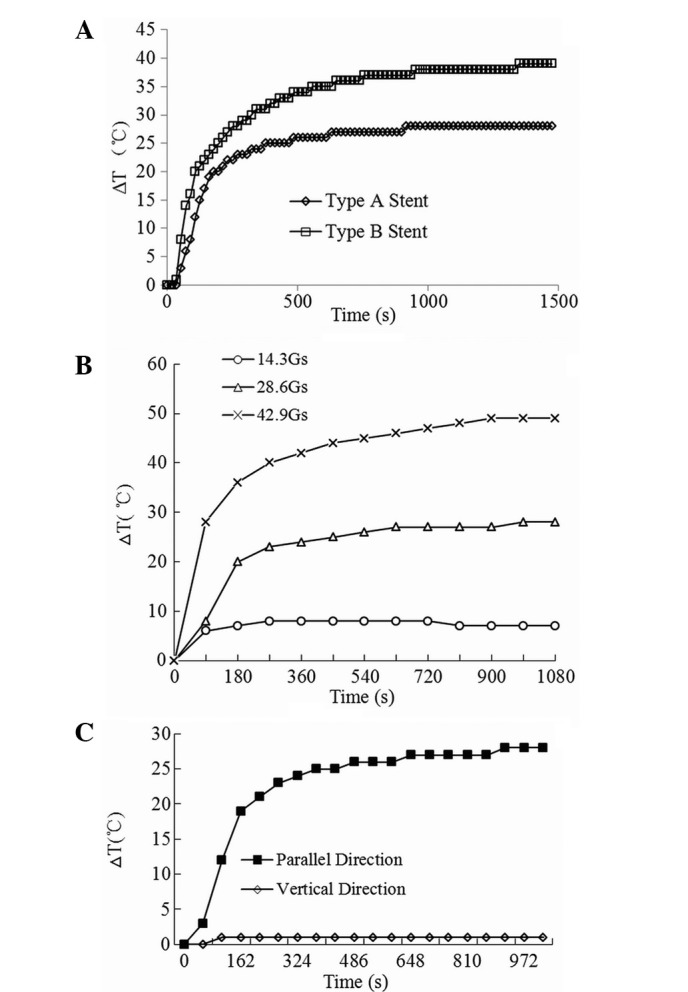
Inductive heating properties of the esophageal stent under an AMF (A) Effect of stent quality with field intensity of 28.6Gs. (B) Effect of field intensity (type B stent). (C) Effect of orientation (type B stent with field intensity of 28.6Gs). Stent quality, field intensity and orientation of stent placement have effect on the inductive heating property of the stents under AMF exposure. AMF, alternative magnetic field.

The inductive heating properties of the two types of esophageal stents under AMF are shown in Fig. 4A. The stents were observed to possess an excellent property for heat generation upon exposure to the AMF. Rapid temperature increases, as indicated by the initial slope of the curves, were observed. Within 200 sec, a high equilibrium temperature was reached in the two stents, with the higher equilibrium temperature occurring in the heavier stent. Compared with the type B stent, the type A stent was softer and more flexible. In order to impose less mechanical injury to the rabbit esophagus by the stent, the *in vivo* experiment was conducted using the type A stent.

The effect of the AMF strength on the heating profile of the stent is shown in Fig. 4B. The field strength was directly correlated with the inductive heating characteristics of the stent; the higher the field strength, the higher temperature at equilibrium. Fig. 4C illustrates the effect of the orientation of the stent axis on the inductive heating. It was clearly revealed that compared with the stent being perpendicular to the direction of the field direction, the stent that was positioned parallel to the field direction induced a higher temperature, thus generating more heat. All the observations strongly indicated that the quality of the stent, the orientation of the stent axis and the field parameter affect the heating profile of the stent under AMF.

**Figure 5:**
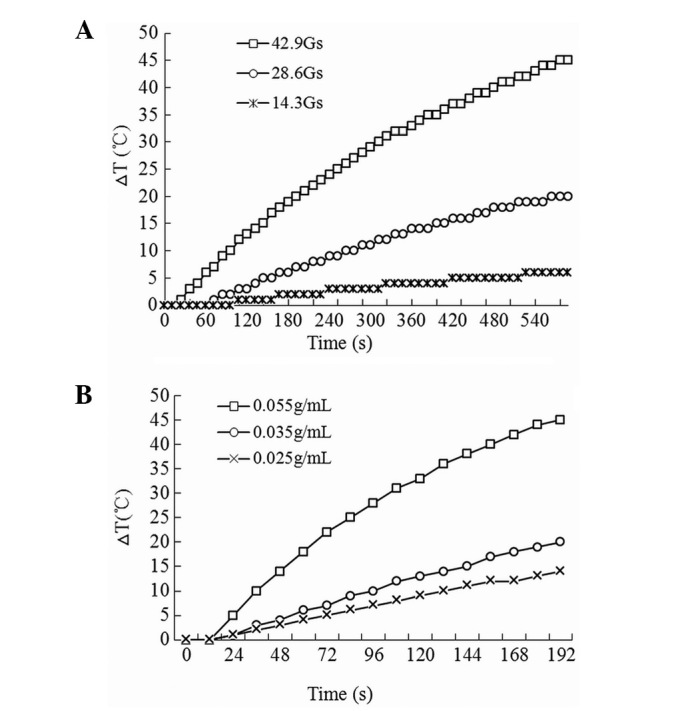
Inductive heating properties of MNPs under an AMF. (A) Effect of field intensity (MNPs concentration of 0.055 g/ml). (B) Effect of MNPs concentration with field intensity of 42.9Gs. MNPs, magnetic nanoparticles; Field intensity of AMF and MNPs concentrations have effect on the inductive heating property of MNPs under AMF exposure. AMF, alternative magnetic field.

The heating profiles of the aminosilane-MNP suspensions with the various MNP concentrations under an AMF of 300 kHz are shown in Fig. 5. A higher field intensity and particle concentration resulted in a greater increase in the temperature. The desired temperature was achieved by appropriately choosing the MNP concentration or adjusting the field intensity, which guaranteed the temperature requirements for the hyperthermia cancer treatment.

### Safety evaluation of MSH on the rabbit esophagus

**Figure 6:**
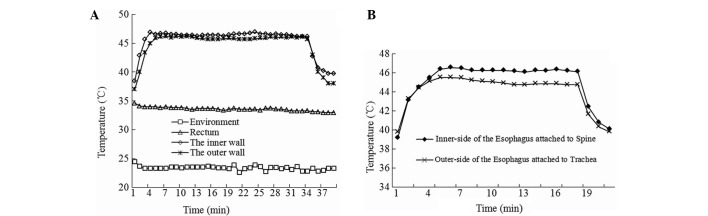
Typical temperature curves of the rabbits under MSH (A) Temperatures measured at the rectum and the inner and outer esophageal walls of the rabbits. (B) Temperatures measured at the inner- and outer-side of the esophagus. MSH, magnetic stent hyperthermia.

Fig. 6 shows the *in vivo* temperature profile of the rabbits under MSH. By carefully adjusting the field parameter, the desired temperatures were achieved and maintained. It was also observed that during the treatment, the rectal temperature was constant, indicating that the MSH was a local treatment. Fig. 6A also shows that there was a slight temperature difference (<2°C) between the inner and outer esophageal walls of the rabbit. The temperature profiles of the various esophagus segments were also examined during the heating process. As shown in Fig. 6B, the inner-side of the esophagus, which is attached to the spine, demonstrated a higher temperature than the outer-side of the esophagus, which is attached to the trachea.

**Figure 7:**
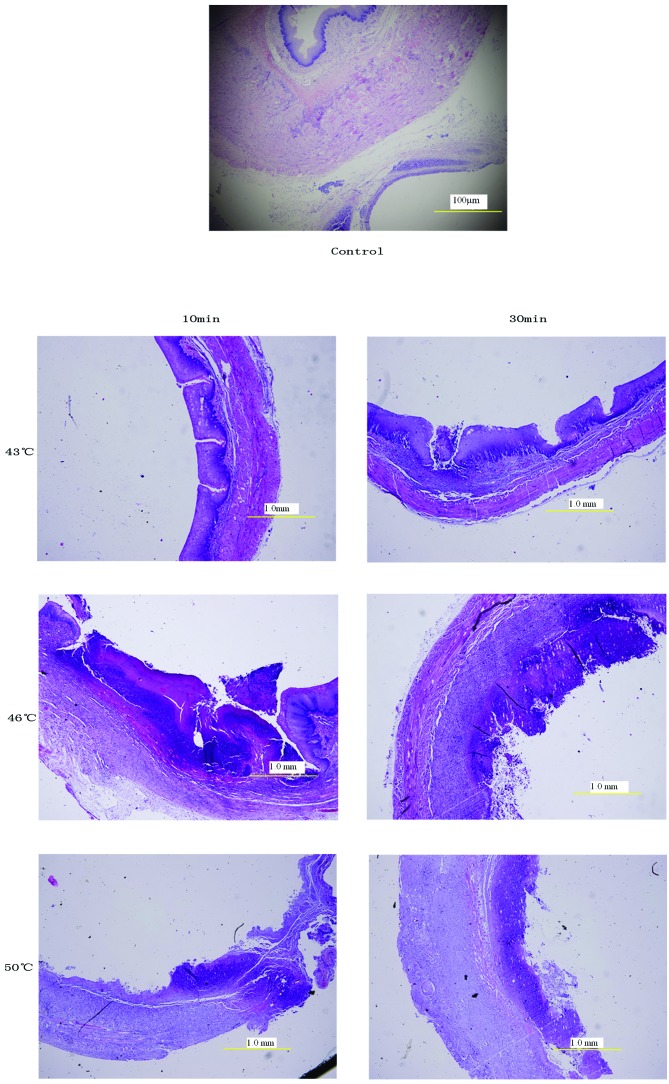
Pathological change of the normal esophagus following magnetic stent hyperthermia (MSH) treatment (HE staining)

The histological evaluation of the rabbit esophagus under MSH of various thermal doses is shown in Fig. 7. Generally, MSH of 50°C was not tolerable to the rabbits, as transmural necrosis occurred in all the animals treated with this temperature. For 46°C hyperthermia, necrosis may reach the submucosa layer due to a longer treatment time (30 min), and for 43°C, the treatment was observed to be safe to the rabbit esophagus, regardless of the treatment time. However, our previous results have demonstrated that esophageal cells are heat resistant (14). A temperature of 43°C has little effect on the viability or necrosis of the ECA-109 cells in the treatment time range of 5–30 min. Relatively, 46°C MSH for 10 min is safe to use on the esophageal wall of the rabbits, as only a slight necrosis was observed in the mucosa layer. Therefore, such a thermal dose was adopted for the antitumor effect evaluation of MSH on the rabbit esophageal tumor model.

### Effect of MSH on esophageal cancer in a rabbit tumor model

**Figure 8:**
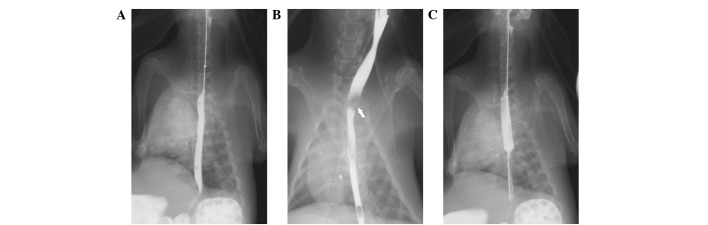
Rabbit esohphageal tumor featured by barium meal and stent implant. (A) Normal rabbit esophagus. (B) Rabbit esohpageal cancer. (C) Implantation of the stent. Arrow, tumor location.

**Figure 9:**
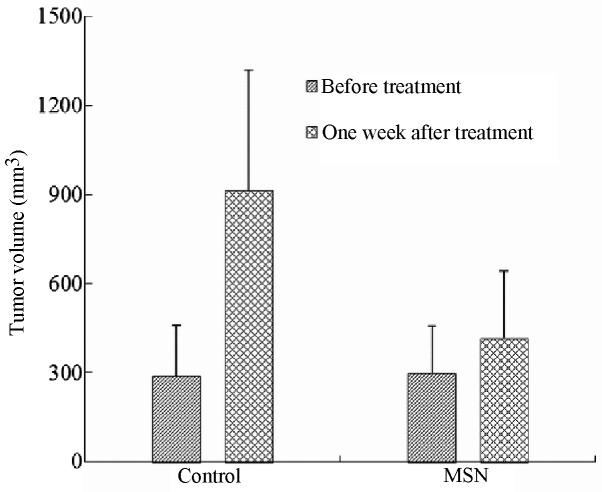
Effect of magnetic stent hyperthermia (MSH; 46°C for 10 min) on tumor volume. MSH using the thermal dose of 46°C for 10 min was able to effectively inhibit the tumor growth in the rabbit esophageal tumor model. MSH, magnetic stent hyperthermia.

Fig. 8 shows the successful establishment of the rabbit esophageal tumor model and implantation of the stent into the rabbit esophagus. Stent migration was not observed in any rabbits during the observation period. Fig. 9 demonstrates the effect of MSH on the tumor volume following one week of the treatment. Prior to the treatment, the tumor volume of the control and treatment groups was 286.3±174.5 and 195.0±162.7 mm^3^ (P>0.05), respectively. One week after MSH, the tumor volume was 415±228.1 mm^3^ for the rabbits under treatment whilst that of the control group reached 913.7±404 mm^3^ (P<0.05). MSH using the thermal dose of 46°C for 10 min was able to effectively inhibit the tumor growth in the rabbit esophageal tumor model.

### Effect of MFH on esophageal cancer in a rabbit tumor model

**Figure 10:**
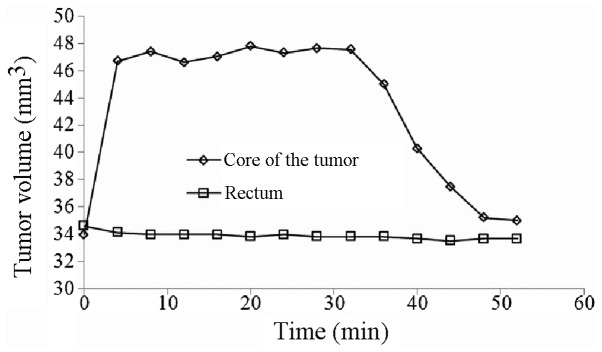
Typical temperature curve of the rabbits undergoing magnetic fluid hyperthermia (MFH). The temperature of the rabbit rectum was kept constant during the treatment, confirming the local treatment of MFH.

**Figure 11:**
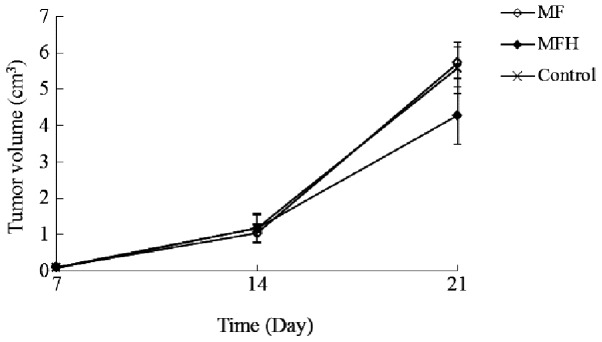
Effect of magnetic fluid hyperthermia (MFH) on tumor volume. MFH can greatly inhibit the in vivo tumor growth.

**Figure 12:**
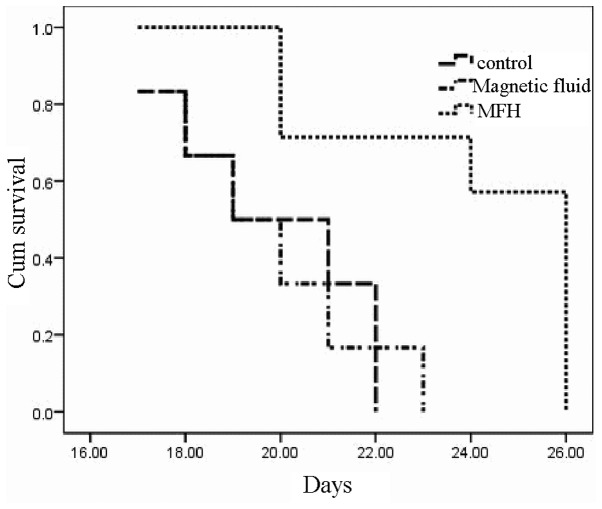
Survival rate of tumor-bearing rabbits in the magnetic fluid hyperthermia (MFH), MF and control groups. MFH was able to significantly increase the life span of the tumor-bearing rabbits over that of the control and MNP injection groups.

**Figure 13:**
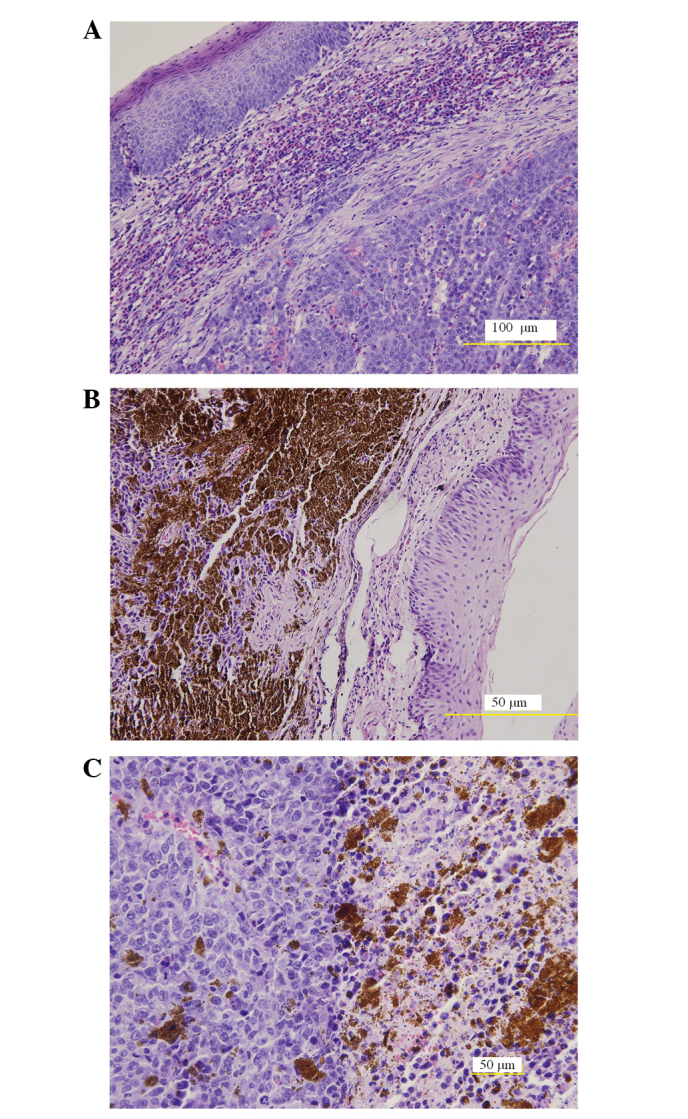
Pathological changes of rabbit esophageal cancer tissue under various treatments (HE staining). (A) Control (B) MF and (C) MFH groups. MFH, magnetic fluid hyperthermia.

Fig. 10 shows the temperature profile of the rabbits that were subjected to MFH. The temperature of the rabbit rectum was kept constant during the treatment, confirming the local treatment of MFH. MNPs also have excellent inductive heating properties *in vivo*. The temperature was able to reach 48°C within 5 min and was stably maintained by carefully adjusting the field parameters. The temperature dropped quickly subsequent to turning the power off, indicating a rapid heat dissipation inside the rabbit. The time course of subjecting the tumor volume to the various treatments is shown in Fig. 11, and demonstrated that the tumor volume of the rabbit in the control group steadily increased with no evidence of regression. By contrast, the injection of the MNPs within the tumor site had no therapeutic effect to the esophageal cancer. However, MFH was able to greatly inhibit the *in vivo* tumor growth (P<0.001). The difference in survival among the three groups is shown in Fig. 12. MFH was able to significantly increase the life span of the tumor-bearing rabbits over that of the control and MNP injection groups. The histological evaluation of the esophageal tumors that were subjected to the various treatments is shown in Fig. 13. MNPs aggregated and distributed within the tumor tissues in the MFH and MF groups. The tumors in the MF group revealed no appearance of necrosis. In the MFH group, the esophageal tumors displayed large areas of necrosis, cell shrinkage and ruptured cell pieces. It was confirmed that no esophagus perforation or tracheoesophageal fistulae occurred in the rabbits in the MFH group, indicating that MFH at 48°C for 30 min is safe for rabbits.

## Discussion

The biological effectiveness of heat in treating cancer has been fully recognized and a number of molecular mechanisms have been elucidated. Since the 1970s, several aspects of heat action have been examined in numerous pre-clinical studies (8). However, an unequivocal identification of the mechanisms leading to favorable clinical results using hyperthermia has not yet been identified. The technical limitations of the heat locoregional delivery and the poor control of the thermal dosage are possible reasons that impede the effective distribution of the therapeutic temperatures and doses in the tumor site, thus restricting the successful application or translation of the research output into clinical oncology. MMH is able to couple the heat magnetically to the mediators or agents only within the tumor site. Generally, as the magnetic mediators are conformably distributed within the tumor site, a homogenous temperature field may be realized. The concept of MMH was first proposed by Gilchrist *et al* in the 1950s, where it was demonstrated that magnetic particles were able to be deposited selectively at the tumor site and heat the tumor tissue specifically when exposed to an AMF (25). Following years of exploration, the research output on MMH has been successfully applied in clinical oncology with inspiring results, thus providing an alternative procedure for cancer treatment.

As the mediator of MMH, the magnetic agents play a critical role in the hyperthermia treatment. The results of the present study confirmed that the stent and MNPs were able to rapidly reach the desired hyperthermia temperature and that the temperature was stably maintained under the proper field parameters. Briefly, the magnetic field induces current to flow in the stent and the resistance of the stent impedes the current flow, thereby producing heat. For the micro- or nano-scaled agents, the mechanisms may be more complicated. However, it has been generally acknowledged that Brownian movement and Neel relaxation mainly account for the inductive heating (26). Although there is a difference between the heating mechanisms, the results of the present study show that the two agents possess excellent inductive heating properties under AMF. The rapid temperature rise is favorable during hyperthermia treatment with regard to thermotolerance (27). More thermotolerance, which may reduce the treatment efficiency, is induced if the temperature rises slowly.

The present study also revealed that the field intensity of the AMF and the quality of the agents were positively correlated with the inductive heating properties. A higher temperature may be achieved with a higher field intensity, heavier stent and higher contents of MNPs within the magnetic suspensions. This observation indicates that during the treatment, the hyperthermic temperature may be controlled by a proper choice of agents or careful adjustment of the field intensity. It was also noteworthy that the orientation of the stents affected the heating profile, and a parallel position of the stent to the field direction produced the highest temperature. However, there was no directional dependence of AMF to the inductive heating property of the nano-scaled or micro-scaled agents. Such a phenomenon may be explained from the difference in the heating mechanisms of the agents.

The *in vivo* heating profile of MSH demonstrated an existing inhomogeneous heat distribution in the esophagus. As shown in Fig. 6, the deep-seated esophagus segment was able to hold more heat than the superficial segment. This is easy to explain from the viewpoint of *in vivo* heat transfer. The present results also demonstrated that the rabbit esophagus was not thermally insulated in the radial direction, as only a slight temperature difference was observed between the temperatures of the inner and outer esophagus segments. This observation is inconsistent with results observed in pigs. In a previous study, a significant difference was observed between the temperatures of the inner and outer esophageal walls of the pig, although an extremely high temperature (>50°C) was reached at the inner esophageal wall, the maximal temperature of the outer wall did not exceed 40°C (14). The thermal conductivity of an organ or a tissue is of vital significance during hyperthermia treatment. A low thermal conductivity may result in a poor heat transfer performance, which is unfavorable for MSH, as the therapeutic effect may be compromised by the thermal insulation of the esophageal wall. Therefore, a thorough understanding of the temperature distribution and heat transfer performance with regard to the esophagus is a priority for the clinical application of MSH.

During either MSH or MFH in the present study, the rectal temperatures of all the rabbits were kept constant during the treatment. Therefore, local hyperthermia was confirmed. Compared with MFH, MSH was able to heat the esophagus segment that was implanted with the stent, as well as the tumor site. As shown in Fig. 7, transmural necrosis was observed when using the thermal dose with a higher temperature or longer treatment time. Safety considerations, particularly heat resistance of the healthy esophagus should thus be carefully evaluated. It should be noted that properties of the human esophagus may not be deduced from observations of the rabbit esophagus, as there may be a difference in the heat resistance and heat transfer performance between humans and rabbits. A similar study was conducted on pigs, with results showing that the pig esophagus was able to endure a higher temperature treatment without mucous hyperemia or tissue edema (14). Although humans and pigs share similar physiological indexes in a number of aspects, it is impossible or inconvenient to grow a tumor in the pig’s esophagus. A rabbit esophageal tumor model may be the only choice to conduct an *in vivo* investigation for MSH in the current situation. Although there may be limitations for the tumor model, the results of the present study have shown that MSH has a positive therapeutic effect under the appropriate thermal dose.

MFH is able to induce heat that is confined within the tumor site. Therefore, more specific heating may be achieved compared with MSH. The results of the present study indicate that MFH at a thermal dose of 48°C for 30 min may effectively inhibit the tumor growth and significantly prolong the life-span of the tumor-bearing rabbits without any harm to the nearby tissue or organs. The present study systematically conducted the effect of various thermal doses on the proliferation and apoptosis of the human esophageal ECA-109 cancer line (13). The observation indicated that the hyperthermia treatment of 48°C for 20–30 min may be the optimal thermal dose. In recognition that a higher temperature and longer treatment time may impose damage to the healthy esophagus if MSH was applied, MFH may be considered safer and more effective than MSH. However, MSH is based on a well-established approach of an endoscopic placement of the stent and therefore other patient procedures are not required, with the exception of AMF exposure. For MFH, the infusion of a colloidal suspension of MNPs within the tumor site is required. In the present study, the MNPs were infused under direct view by an open surgical exposure of the esophagus. In order to achieve a mini-invasive or non-invasive surgery when applied in clinical oncology, a special infusion device should be developed for the administration of the MNPs perorally. Therefore, MSH may be more convenient for clinical translation. Furthermore, hyperthermia is usually applied as an adjunct to an established treatment modality and aims at improving the results of the conventional treatment strategies within the framework of multimodal treatment concepts. Therefore, an improved therapeutic effect may be achieved by the combination of MSH and radiotherapy or chemotherapy. Akiyama *et al* performed pioneering clinical work on the feasibility and effectiveness of the multimodal treatment of MSH combined with simultaneous chemotherapy in 13 patients and radiochemotherapy in five patients (11). The results indicated that MSH proved effective in eight of the nine patients who were administered the treatment at least three times. The clinical results indicated that MSH is able to improve the effectiveness of combination therapy and suppress local tumor growth. Therefore, it is suggested that while MFH may be applied as a monotreatment for esophageal cancer, MSH may be more suitable for multimodal treatment by combining hyperthermia with other cancer treatment approaches.

MSH and MFH promise to be local and effective hyperthermia treatments for esophageal cancer. MFH is able to induce heat within the tumor site only, in order to achieve more specific heating, and therefore, may be applied as a monotreatment for esophageal cancer. The homogenous local infusion of colloid MNPs with an ideal inductive heating property is required for MFH. MSH is able to combine the advantages of stent endoscopic placement with local heating. The clinical stent possesses an excellent inductive heating property under an AMF, and therefore, there is no requirement for the development or administration of other devices for MSH. However, a thorough understanding of the heat resistance and heat transfer performance of the human esophagus is of vital importance to facilitate the logical transition of the technique from the bench to the bedside.
